# Supplementary material for: A set of multi-entry identification keys to African frugivorous flies (Diptera, Tephritidae)
Source: Zookeys. 2014 Jul 24;(428):97–108. doi: 10.3897/zookeys.428.7366 (PMC4143993; doi:10.3897/zookeys.428.7366)
Supplement: Supplementary material 4 — Key to Capparimyia [file zookeys-428-097-s004.zip › SF4_ZooKeys_key to Capparimyia/lucid_player/help/help/how_to_check_if_you_have_java_installed.htm]

How to check if you have Java installed


# How to check if you have Java installed

Java Runtime Environment (JRE) version 1.4.2 or greater must be installed
for Lucid3 to run successfully. To check if you have Java installed on
your system, type:

Java -version

 in a
Command prompt (Windows) or Terminal console (Mac, Unix).

 For example:

If you receive an �Unknown Command� error message or your Java build
number is less than 1.4.2 then you need to install the latest (free) edition
of JRE, which can be found at www.java.com

For help installing the JRE please see: www.java.com/en/download/help/index.xml

For FAQ on the JRE please see: www.java.com/en/download/faq/index.xml
